# Geochemical wolframite fingerprinting – the likelihood ratio approach for laser ablation ICP-MS data

**DOI:** 10.1007/s00216-018-1007-9

**Published:** 2018-04-17

**Authors:** Agnieszka Martyna, Hans-Eike Gäbler, Andreas Bahr, Grzegorz Zadora

**Affiliations:** 1 Department of Analytical Chemistry, Institute of Chemistry, The University of Silesia, Szkolna 9, 40-006 Katowice, Poland; 2 Federal Institute for Geosciences and Natural Resources (BGR), Stilleweg 2, 30655 Hannover, Germany; 3 Institute of Forensic Research, Westerplatte 9, 31-033 Krakow, Poland

**Keywords:** Wolframite, Fingerprinting, Laser ablation ICP-MS, Likelihood ratio approach, Chemometrics

## Abstract

Wolframite has been specified as a ‘conflict mineral’ by a U.S. Government Act, which obliges companies that use these minerals to report their origin. Minerals originating from conflict regions in the Democratic Republic of the Congo shall be excluded from the market as their illegal mining, trading, and taxation are supposed to fuel ongoing violent conflicts. The German Federal Institute for Geosciences and Natural Resources (BGR) developed a geochemical fingerprinting method for wolframite based on laser ablation inductively coupled plasma-mass spectrometry. Concentrations of 46 elements in about 5300 wolframite grains from 64 mines were determined. The issue of verifying the declared origins of the wolframite samples may be framed as a forensic problem by considering two contrasting hypotheses: the examined sample and a sample collected from the declared mine originate from the same mine (H_1_), and the two samples come from different mines (H_2_). The solution is found using the likelihood ratio (LR) theory. On account of the multidimensionality, the lack of normal distribution of data within each sample, and the huge within-sample dispersion in relation to the dispersion between samples, the classic LR models had to be modified. Robust principal component analysis and linear discriminant analysis were used to characterize samples. The similarity of two samples was expressed by Kolmogorov-Smirnov distances, which were interpreted in view of H_1_ and H_2_ hypotheses within the LR framework. The performance of the models, controlled by the levels of incorrect responses and the empirical cross entropy, demonstrated that the proposed LR models are successful in verifying the authenticity of the wolframite samples.

**Electronic supplementary material:**

The online version of this article (10.1007/s00216-018-1007-9) contains supplementary material, which is available to authorized users.

**Graphical abstract:**
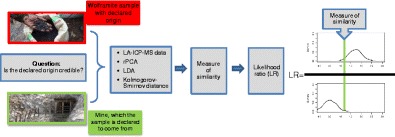
Geochemical wolframite fingerprinting

## Introduction

In the eastern provinces (North Kivu, South Kivu, and Maniema) of the Democratic Republic of the Congo (DRC), ongoing violent conflicts are fuelled by illegal mining, trading, and taxation of natural resources (e.g., tin, tantalum, and tungsten, their ores, and gold). Foreign and local armed groups profit from mining activities and use the revenue from mineral trade to finance their troops [1, 2]. In 2010 the US Congress passed the Dodd-Frank Wall Street Reform and Consumer Protection Act and charged the Securities and Exchange Commission (SEC) to take action to address virtually all of the mandatory rulemaking provisions of the Act. Section 1502 of this Act requires US-listed companies to exercise due diligence on the traceability of so-called “conflict minerals” (coltan, cassiterite, and wolframite mined to obtain Ta, Sn, and W, respectively, and gold) or their derivatives originating from DRC or adjoining countries if these minerals are necessary for the functionality or production of their products [3]. On the one hand, the Dodd-Frank Act intends to reduce income from mineral trade for armed groups, but on the other hand this Act will also have great impact on regular artisanal miners whose livelihood is strongly dependent on mining of these minerals. However, recently a combination of court opinions, regulatory reversals, and legislative proposals have joined to weaken the conflict mineral regulations under Section 1502 [4]. In 2017, the European Parliament and the Council laid down supply chain due diligence obligations for Union importers of tin, tantalum, and tungsten, their ores, and gold originating from conflict-affected and high-risk areas [5].

Traceability systems for mineral supply chains are designed to (1) indicate shipments which are of reliable origin and not conflict affected, and (2) to hamper market access for illegally mined and traded ores. Within such systems each ore mineral shipment is accompanied by a document which provides information about the origin of the minerals. An analytical fingerprinting (AFP) approach has been developed at the German Federal Institute for Geosciences and Natural Resources (BGR) as a document-independent tool to verify the declared origin of a shipment in case of doubt [6–8]. AFP can be implemented as an optional proof of origin within the framework of traceability systems.

For AFP, a sample is taken from a shipment in doubt, the sample is analyzed, and the results are evaluated by comparison with data from a reference sample database where mine-specific information on ore minerals is stored. The result is a statement whether the documented origin of the shipment in doubt is credible or not.

Wolframite (Fe,Mn)WO_4_ is the most important ore mineral for tungsten in Central Africa. Tungsten is a metal of high economic importance with major applications in cutting tools as tungsten carbide, in the production of various steel grades as an alloying component, or as filaments in light bulbs. Wolframite is traded as an ore concentrate which is produced by miners at the mine site.

Recently, Gäbler et al. [8] presented an approach for the analytical fingerprinting of wolframite ore concentrates based on laser-ablation inductively coupled plasma-mass spectrometry data, the evaluation of Kolmogorov-Smirnov distances of two-sample comparisons, and an empirically derived decision criteria. The data from wolframite concentrates are multivariate, not normal-distributed, and due to the mining process samples cannot be regarded as representative aliquots of a population, which poses an additional challenge for data evaluation [8]. This study presents an alternative data evaluation approach based on the likelihood ratio concept (e.g., [9–11]) and is based on the nearly identical data set used by Gäbler et al. [8].

To confirm or dispel the doubt that arises concerning a sample’s origin, the following question must be considered – does the sample under investigation originate from the declared mine? Then, if (i) E stands for a sample under investigation which is declared to come from source S (i.e., location, mine site), and (ii) D stands for a reference sample truly coming from this declared origin S, then the proposed methodology addresses the forensic comparison problem [9–12] in which two competing hypotheses are stated:

- H_1_ - samples D and E come from the same source S, i.e., mine site,
- H_2_ - samples D and E originate from different sources.

The problem in practice boils down to verifying whether D and E samples are so-called brother samples (samples sharing a common origin) or not. Then such a comparison issue may be simplified by grounding it in the classification task [13, 14] in which the following hypotheses are investigated:

- H_1_ - samples D and E are brother samples,
- H_2_ - samples D and E are not brother samples.

One of the solutions of this issue requires comparing the similarity of samples E and D with the similarity of sample E and each individual sample X remaining in the reference database based on the samples elemental composition. First, the characteristic of samples D and X is derived by a chemometric procedure (robust principal component analysis (rPCA) combined with linear discriminant analysis (LDA), details are given below) recording the difference between them. Now the data of sample E are projected on the variable characterizing and differentiating samples D and X. The idea is that if samples D and E are brother samples, both samples should behave similar relative to each individual sample X from the reference sample database and not similar if they are not brother samples. The final conclusive stage involves deciding whether this similarity of samples E, D, and X is more likely to occur when E and D are brother samples (H_1_) or when they are not (H_2_). Such a problem raised in the perspective of two equivalent hypotheses, H_1_ and H_2_, typically issued in the forensic sciences, should preferably be solved using the likelihood ratio theory of hypothesis testing [9]. The equivalence of both hypotheses stated in the LR approach remains in contrast to the willingly applied statistical tests (e.g., *t*-test), in which the hypotheses are not equiponderant. These tests only indicate whether the null hypothesis (on which the emphasis is put) is rejected or fails to be rejected. No conclusions can be made about the acceptance/rejection of the alternative hypothesis.

For discrete measurements, the probability that evidence (*ε*) characterized by variable *Z* takes the value *z* if H_1_ is true is denoted Pr(*Z* = *z*|H_1_). Similarly, Pr(*Z* = *z*|H_2_) denotes the probability that *Z* takes the value *z* when H_2_ is true. The likelihood ratio compares the probability that *Z* = *z* when H_1_ is true with the probability that *Z* = *z* when H_2_ is true (Equation 1).

1 $$ LR=\frac{\Pr \left(Z=z|{H}_1\right)}{\Pr \left(Z=z|{H}_2\right)}=\frac{\Pr \left(\varepsilon |{H}_1\right)}{\Pr \left(\varepsilon |{H}_2\right)} $$

LR measures the strength of the evidence in favor of H_1_ compared with H_2_ when *Z* = *z*. For continuous measurements, similar reasoning holds with the probabilities replaced by probability density functions *f*(*Z=z|*H_1_) and *f*(*Z=z|*H_2_):

2 $$ LR=\frac{f\left(Z=z|{H}_1\right)}{f\left(Z=z|{H}_2\right)}=\frac{f\left(\varepsilon |{H}_1\right)}{f\left(\varepsilon |{H}_2\right)} $$

The likelihood ratio is not a probability but a ratio of probabilities, and hence it takes values between 0 and infinity. Values of the likelihood ratio above one support the H_1_, the values below one support the H_2_, and those equal to one support neither of the hypotheses. The higher the value of the likelihood ratio is, the stronger is the support for the H_1_ proposition. The lower the value of the likelihood ratio is, the stronger is the support for the H_2_ proposition.

Another advantage of the LR approach over other statistical tests is the consideration of the rarity of the samples’ data. This rarity is available from databases storing information about the same parameters measured for a representative set of samples. Observing similar features for both compared samples must always be carefully controlled as the match between characteristics may be just a coincidence. This danger is growing for features commonly observed in the relevant population and decreases with their increasing rarity. Thus the value of the evidence in support of the proposition that compared samples have common origin is greater when the determined values are similar and rare in the relevant population than when the physicochemical values are equally similar but common in the same population [9, 11]. The rarity considerations are unfortunately ignored in the score-based LR models, where the similarity between characteristics of two samples is expressed by their distance. Since the distance is identically measured for rare and common data, the score-based LR models’ virtue mainly boils down to computational efficiency. Nevertheless, the score-based LR models still keep their superiority over other statistical tests by viewing the data from two equivalent contrasting perspectives (hypotheses).

LR is a method for commenting on the evidential value of the evidence material, which is recommended by the forensic community, including the European Network of Forensic Science Institutes [15–19]. The most successful application of the LR approach in the forensic sphere is found in the evaluation of DNA profiling for forensic purposes [20]. This approach has also been used in the analysis of earprints, fingerprints, firearms, and tool marks, hair, documents, and handwriting (review can be found in [9]), as well as speaker recognition [21]. An increasing number of applications of this approach is found in the evaluation of physicochemical data recorded for microtraces of glass [12–14, 22–27], explosives [28], car paints [29–33], polymers [31, 32], fire debris [34], inks [35, 36], fibers [29], drugs [37–39], food samples [40, 41] and biological samples [42].

Since the work of Aitken and Lucy [10] was published, LR models have been widely developed for data sets described by a limited number of variables. Commonly analyzed evidence in the form of glass fragments characterized by their elemental composition [12–14, 22, 23] concerning only oxygen, sodium, magnesium, aluminium, silicon, potassium, calcium, and iron, may serve as an example. Similar to most of the statistical methods, classic, so-called feature-based LR suffers from the *curse of dimensionality* when dealing with highly multidimensional data, being currently a domain of most of the analytical techniques outcomes. Moreover, difficulties emerge when the data are not normally distributed within each sample and their variance structure becomes complex. This may be the case when dispersion of data within each sample and for the samples from the same source (e.g., mine site) is comparable to the dispersion of data for samples from different sources. Some strategies for dealing with the multidimensionality have been proposed in [31, 32] for infrared and Raman spectra. They engage chemometric tools for reducing data dimensionality by studying various sources of variability and extracting the most relevant information in the form of a few latent variables. The outcomes of the chemometric techniques are then incorporated in what is referred to as hybrid LR models [31, 32]. The issues of the lack of normality and significant within-sample data dispersion have not been tackled yet. However, some strategies have been studied recently for keeping the proper relation of the within- and between-samples variability, which is easily violated by the applied chemometric tools for reducing data dimensionality.

The multidimensionality and lack of data normality within each sample is not regarded to be an obstacle in the score-based LR models. These models maximally reduce data dimensionality to only a single score describing two compared samples. The score, which is for instance the distance between two samples characteristics, is then interpreted in the light of two hypotheses, H_1_ and H_2_. In the score-based LR models constructed for the examined wolframite data, the score is the similarity metric between the questioned sample, the sample from the declared mine site, and each of the remaining samples collected in the database. These similarity metrics must be significantly different for brother and non-brother samples. This is possible only when the distances are computed in the space defined by the variables that well differentiate between samples from different locations and effectively group brother samples. Thus the dispersion of data for brother samples should be kept much lower than for non-brother samples. This is easily achieved using chemometric tools optimally separating classes (or samples if each sample is regarded as a class), such as linear discriminant analysis (LDA). The only requirements of LDA are the reduction of data dimensionality and the need to deal with a non-normal distribution within each sample. Even then, when care must be taken to work with normally distributed data and reduce their dimensionality, the use of score-based LR models is not purposeless. This is because scores provide an improved description of the similarity between samples and consequently better enable the decision of whether they are brothers or not than conventional, feature-based LR models.

Thus the aim of this work is to demonstrate that hybrid score-based likelihood ratio models are capable of verifying the authenticity of wolframite concentrate origins declared in the official documents. The issue is tackled with the combination of chemometric tools and the LR approach in the form of hybrid LR models [31, 32]. They utilize various chemometric techniques for (1) reducing data dimensionality, and (2) dealing with different aspects of database structure, i.e., lack of normality and significant dispersion arising from huge ranges of elements content observed within each sample and between them. The models engaged (i) robust variant of principal component analysis for reducing data dimensionality [43–45], (ii) linear discriminant analysis (LDA) [43] for finding the directions that capture the differences between samples, and (iii) Kolmogorov-Smirnov distance [46] for expressing their similarity, which, as a score, was then viewed within the LR framework.

## Materials and methods

### Samples, sample preparation, and analysis

Throughout this study, a sample is referred to as an aliquot of an ore concentrate which contains several hundred or several thousand individual mineral grains. The majority of those grains are wolframite grains if a good ore concentrate is obtained. Sample properties in terms of distributions of element concentrations in wolframite are obtained from about 40 to 50 individual wolframite grains of a sample.

For analysis a polished section is prepared for each sample. Wolframite grains are identified by scanning electron microscopy and analyzed by laser-ablation inductively coupled plasma-mass spectrometry. Details on sample preparation, grain identification, and grain analyses are given by Gäbler et al. [8].

The database used for this study consists of information on elemental composition of 104 wolframite samples and is nearly identical to the database used by Gäbler et al. [8]. The wolframite ore concentrate samples originate from 45 different mine sites from 10 countries worldwide, with special emphasis on Central Africa (30 mine sites). In total, 5327 wolframite grains have been analyzed for the elements Mg, Ca, Sc, Ti, V, Cr, Mn, Fe, Co, Ni, Cu, Zn, Ga, As, Sr, Y, Zr, Nb, Mo, Ag, Cd, In, Sn, Sb, Ba, La, Ce, Pr, Nd, Sm, Eu, Gd, Tb, Dy, Ho, Er, Tm, Yb, Lu, Hf, Ta, Tl, Pb, Bi, Th, and U. There were 105 pairs of brother samples (samples coming from the same mine site) and 4972 pairs of non-brother samples (samples coming from different mine sites).

### LR models construction protocol

**Fig. 1:**
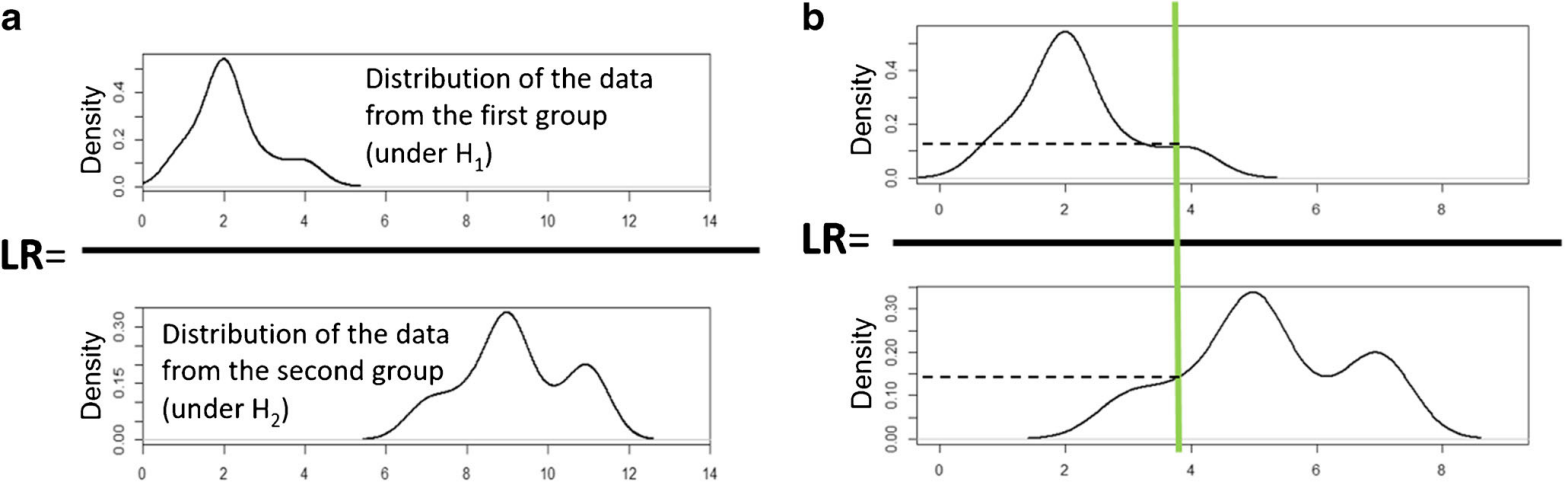
The idea of using score-based LR models in **(a)** an ideal situation when distributions in the numerator and denominator are separate, and **(b)** real situation when the distributions partial overlay. The green line demonstrates the way data should be interpreted in the context of both distributions in the numerator and denominator considered under hypotheses H_1_ and H_2_

The problem of wolframites authenticity was investigated by considering two hypotheses, H_1_ (E and D are brother samples) and H_2_ (E and D are not brother samples). The idea of evaluating the similarity of E, D, and X samples in the context of the two hypotheses is visually presented in Fig. 1. As it is displayed there, the probability density of observing a particular similarity metric between samples E, D, and X (illustrated by a vertical solid green line in Fig. 1) is estimated for numerator and denominator, i.e., in the context of the distributions representing the similarity values observed when E and D are brother samples (H_1_) and when they are not (H_2_). Then both probability density values are compared by taking their ratio, which is known as the likelihood ratio (Equation 2).

Score-based LR models successfully distinguish samples only if the characteristics among brother samples are much less dispersed than the characteristics between non-brother samples. As will be evidenced in “Descriptive statistics” section, the dispersion of the data within brother samples is for many elements basically comparable to the dispersion of data observed for the non-brother samples. Moreover, the distributions of data within each sample cannot be considered normal and the number of variables needs to be reduced. Thus the key to build the appropriate LR models for making inferences whether the samples are brothers or not is first by reducing data dimensionality and dealing with the lack of normality, and second by finding the most informative variables with the best discrimination power, which uniquely characterize each mine site and well differentiate each from the others. Thus maximizing the similarity of the brother samples and minimizing the similarity of the non-brother samples is of crucial importance.

First, the original variables were log-transformed for reducing huge data ranges (even 6 orders of magnitude). Then robust PCA (rPCA) [43–45] was applied with the aim of data mining to explore and find patterns in a multivariate dataset containing many extreme values. Its principle is to expose such projections of the original data that maximize their variation in a few components and hence reduce the number of variables. In rPCA robust measures of location and dispersion (namely median and median absolute deviation (MAD) [43]) are used to autoscale the data so that the variables introduce equal amount of variation and neither is favored. The autoscaling formula is expressed as *z*_*ij*_ = (*x*_*ij*_ − *median*(*x*_*i*_))/*MAD*(*x*_*i*_) where: *x*_*ij*_ is the *j*-th observation of *i*-th variable; *median(x*_*i*_*)* and *MAD(x*_*i*_*)* are the median and median absolute deviation of the *i*-th variable. The utmost advantage of the algorithms for rPCA is that they seek for the directions along which the robust measure of spread (MAD) is maximized. This ensures that the creation of the PCA space is minimally affected by extreme values since robust measures of dispersion are resistant to them.

Even though in many cases PCA is reported as sufficient for visualizing data and finding the grouping patterns, the method, when applied to the entire database, was much more successful in catching the significant within-samples variability instead of the variability responsible for the differences between samples. Consequently, the first few PCs carrying the highest part of variability usually did not address the part of information associated with the discrepancies between samples as illustrated schematically in Fig. S1 in the Electronic Supplementary Material (ESM). Thus, instead of applying the rPCA to the entire database, it was then used for reducing data dimensionality for pairs of samples D and each of its non-brother samples available in the database (X_f_, with f = 1 to *k*_*D*_, *k*_*D*_-number of non-brother samples of D in the database) to the number of components explaining 95% of MAD^2^. Thanks to this treatment it was easier to handle the problem with huge dispersion within and between samples for a pair of samples than for the entire database.

**Fig. 2:**
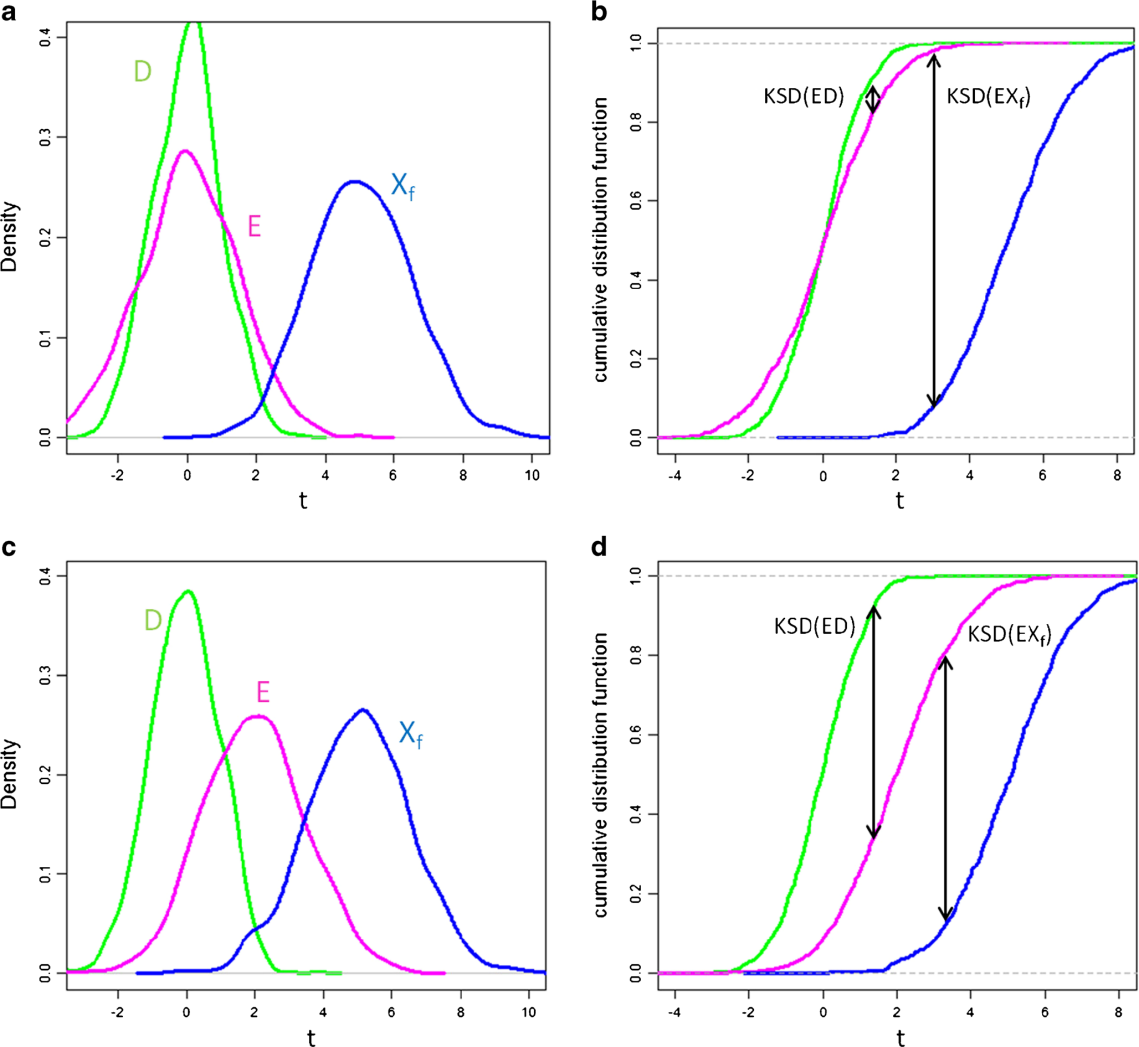
Illustration of the distributions of E, D, and X_f_ samples on the LDA direction (*t*) when **(a)** D and E are brother samples, and **(c)** D and E are not brother samples. The corresponding Kolmogorov-Smirnov distances (KSD) are given in **(b)** and **(d)**, for details see text

Second, LDA [43] was applied for locating the direction that successfully finds the differences between samples D and X_f_ coming from different mine sites. The data of samples D, X_f_, and E were projected on the developed PCA directions and then on the LDA direction (*t*) as shown in Fig. 2. The idea is that if samples D and E are brother samples, both samples should behave similar relative to each individual sample X_f_ from the reference sample database and not similar if they are not brother samples.

The similarity of the distributions of projections of E, D, and X_f_ samples was studied by computing the Kolmogorov-Smirnov distance between the distributions for E and D [KSD(ED)] and between the distributions for E and X_f_ [KSD(EX_f_)] as shown in Fig. 2. The Kolmogorov-Smirnov distance is given as the maximum distance between two cumulative distribution functions (Fig. 2b and d). In the wolframite context the KSD(ED) values are supposed to be low for brother samples [see KSD(ED) distance between E and D samples in Fig. 2b], whereas for non-brother samples they should demonstrate higher values (Fig. 2d). Finally, each set of samples D, X_f_, and E was characterized by the ΔKSD defined as ΔKSD = $\Delta {\mathrm{KSD}}_{{\mathrm{EDX}}_{\mathrm{f}}}$ = KSD(ED) – KSD(EX_f_). Their expected values are listed in Table 1.

**Table 1:** Possible configurations of brother samples (B) and non-brother samples (nB) and the Kolmogorov-Smirnov distance (KSD) values they generate

Case	D and X_f_^a^	D and E ^a^	X_f_ and E ^a^	KSD(ED)^b^	KSD(EX_f_) ^b^	ΔKSD ^b^	Considered under
I	B	B	nB	impossible			-
II	B	nB	B	impossible			-
III	nB	B	B	impossible			-
IV	B	B	B	↓	↓	~0	-
V	nB	nB	nB	↑	↑	~0	H_d_
VI	nB	nB	B	↑	↓	>0	H_d_
VII	nB	B	nB	↓	↑	<0	H_p_
VIII	B	nB	nB	↑	↑	~0	-

^a^ D – sample from the declared mine site, E – sample with questioned origins, X_**f**_ – any other sample from the reference database;

^b^ KSD(ED), KSD(EX_**f**_), ΔKSD – Kolmogorov-Smirnov distances and their difference (for explanations see “LR models construction protocol” section)

For a single case when the source of sample E is declared as common with the location of sample D, *k*_*D*_ ΔKSD values ($\Delta {\mathrm{KSD}}_{{\mathrm{EDX}}_1}$, ..., $\Delta {\mathrm{KSD}}_{{\mathrm{EDX}}_{\mathrm{kD}}}$) were produced. All these *k*_*D*_ ΔKSD values must be integrally and globally interpreted in the context of H_1_ and H_2_ for commenting whether E and D come from the same source or not. Unfortunately, incorporating all *k*_*D*_ ΔKSD values at once for producing a single LR value is not feasible since the LR is computed only for a single value (as in Fig. 1); hence, each value generates a single LR. Thus, dealing with a set of *k*_*D*_ ΔKSD either results in receiving *k*_*D*_ LR values or in one LR value when all *k*_*D*_ ΔKSD are somehow aggregated in a single number and subsequently interpreted within the LR framework.

**Fig. 3:**
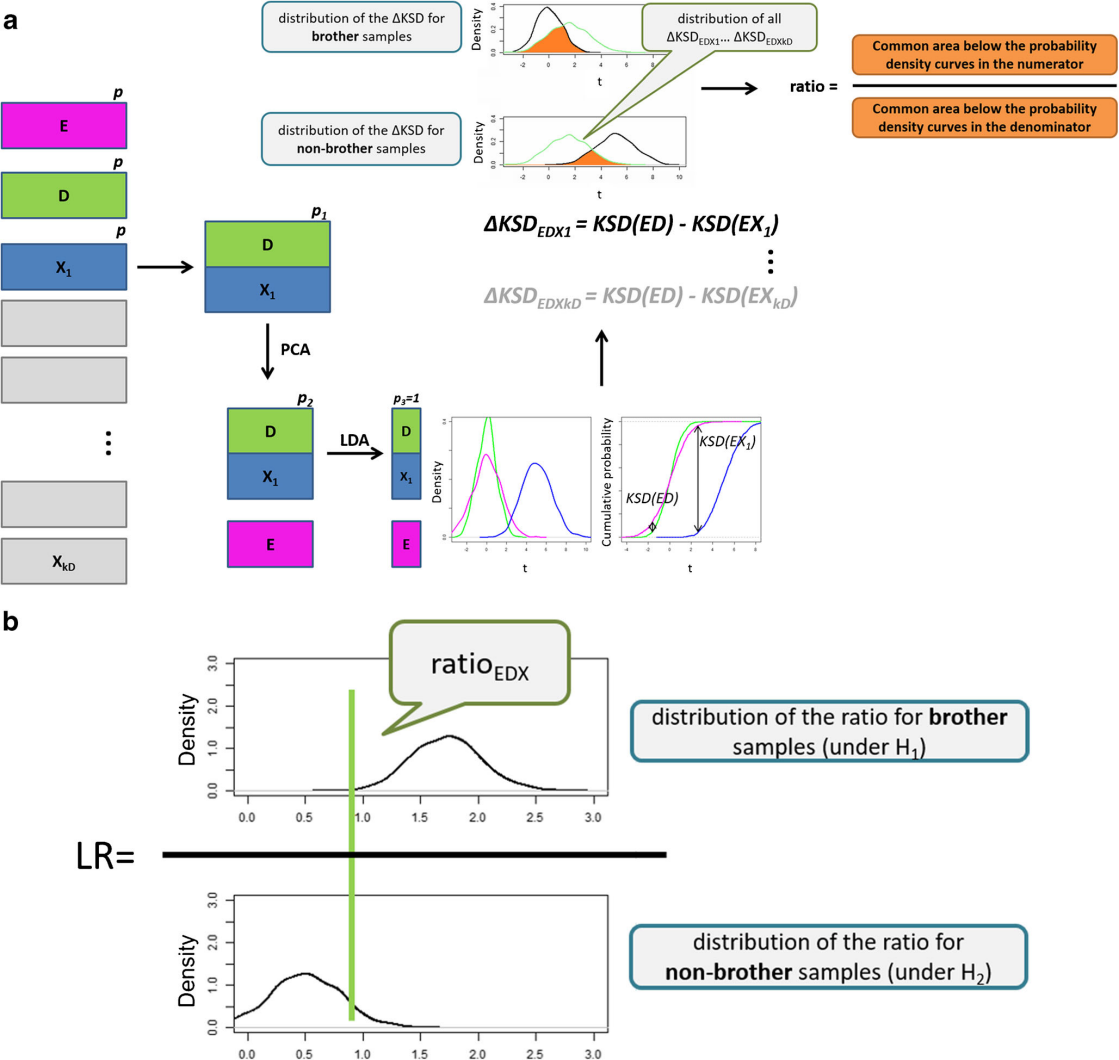
The scheme presenting the idea of **(a)** ΔKSD-AR and **(b)** ΔKSD-AR-LR models. ΔKSD-AR-LR model bases on computing the common areas ratios as in **(a)**, and incorporating them in the LR framework as in **(b)**

The latter idea was tackled in two approaches illustrated in Fig. 3. They are both found in analogy to the conventional problem of computing LR for a single value. This analogy is put forward in computing the common areas of:

- the distribution of ΔKSD for random selection of brother samples (distribution considered under H_1_) and the distribution of *k*_*D*_ ΔKSD obtained for the studied set of D, E and *k*_*D*_ samples X (Fig. 3a),
- the distribution of ΔKSD for random selection of non-brother samples (distribution considered under H_2_) and the distribution of *k*_*D*_ ΔKSD obtained for the studied set of D, E, and *k*_*D*_ samples X (Fig. 3a).

In the first model, referred to as ΔKSD-AR (Fig. 3a), the ratio of both areas (AR) was computed to indicate which of the hypotheses is supported. It should exceed 1 when E and D are brother samples and should remain below 1 for non-brother samples. Though it may appear that this is an LR approach, it is not. This is a consequence of the fact that conventional LR models are computed as a ratio of probability density functions, not the areas below the probability density curves. Thus the proper LR model (denoted as ΔKSD-AR-LR; Fig. 3) was developed in which the sets of common areas ratios received when E and D samples are brothers and when they are not, are stored to find the distributions under H_1_ and H_2_, respectively. Then for the studied set of E and D samples and all *k*_*D*_ X samples the areas’ ratio is computed and interpreted in the context of the modeled distributions under H_1_ and H_2_.

The distribution of common areas ratio studied under H_1_ for numerator and H_2_ for denominator cannot be assumed normal, hence kernel density estimation [47] was used for modeling the underlying distributions. For ΔKSD-AR-LR model the equation reads as follows [9, 11]:

3 $$ \mathrm{LR}=\frac{f\left(\mathrm{AR}|\mathrm{AR}\ \mathrm{values}\ \mathrm{under}\ {\mathrm{H}}_1\right)}{f\left(\mathrm{AR}|\mathrm{AR}\ \mathrm{values}\ \mathrm{under}\ {\mathrm{H}}_2\right)}=\frac{{\left({h_1}^2{c^2}_1\right)}^{\hbox{-} 1/2}\frac{1}{m_1}\sum \limits_{i=1}^{m_1}\exp \left(\hbox{-} \frac{1}{2}{\left(y\hbox{-} {x}_{1i}\right)}^2{\left({h_1}^2{c^2}_1\right)}^{\hbox{-} 1}\right)}{{\left({h_2}^2{c^2}_2\right)}^{\hbox{-} 1/2}\frac{1}{m_2}\sum \limits_{i=1}^{m_2}\exp \left(\hbox{-} \frac{1}{2}{\left(y\hbox{-} {x}_{2i}\right)}^2{\left({h_2}^2{c^2}_2\right)}^{\hbox{-} 1}\right)} $$

Where: *y* - the common areas ratio (AR) under assessment for E, D, and *k*_*D*_ X samples, *c*^2^_1_, *c*^2^_2_ - variances of the *m*_1_ and *m*_2_ common areas ratios (iterated *x*_1*i*_, *x*_2*i*_) considered under H_1_ for numerator and H_2_ for denominator, respectively, *h*_1_, *h*_2_ - smoothing parameter for a single variable (*p* =1) ${h}_g={h}_{opt}={\left(\frac{4}{m_g\left(2p+1\right)}\right)}^{\frac{1}{p+4}}$, (*g*=1 – for numerator, 2 – for denominator) [47].

### Measure of performance

#### Validation scheme

Separate sets of training data for building up the rPCA space, finding LDA direction (*t*), and for modeling the ΔKSD distributions were implied. It is worth emphasizing that the training sets are composed of randomly selected grains from each sample. Thus the dispersion of the data subset after the random selection is kept at the same level as observed for the entire database.

The process of model construction and testing its performance is repeated for several training and test sets. The procedure is applied for averaging the results and making the conclusions resistant and robust towards the cases when the selection of the grains is not representative enough and delivers extremely high or low rates of false responses.

For ΔKSD-AR model, two datasets are required:

- set A consisting of 2*b* pairs of D and E samples (*b* when E and D are brothers and *b* when they are not), each pair with *k*_*D*_ X_f_ samples (Table 1), for computing 2*b∑k*_*D*_ ΔKSD values. These ΔKSD values are used for modeling the distributions when E and D are brothers and when they are not (black distributions in Fig. 3a, each composed of *b∑k*_*D*_ ΔKSD values);
- set B consisting of 2*Z* pairs of D and E samples (*Z* when E and D are brothers and *Z* when they are not), each pair with *k*_*D*_ X_f_ samples (Table 1), for computing 2*Z∑k*_*D*_ ΔKSD values; 2*Z* sets of *k*_*D*_ ΔKSD values each for 2*Z* pairs of E and D samples will be used for computing 2*Z* common areas ratios (AR) with distributions of set A when E and D are brothers and when they are not. The distribution of *k*_*D*_ ΔKSD values for one of 2*Z* pairs of E and D samples is shown in green in Fig. 3a. Then the areas taken for computing ratios are illustrated in orange in Fig. 3a. These AR values are used for estimating the levels of false positive answers (when AR should be lower than unity but it demonstrates values above 1) and false negative rates (when AR should exceed unity but it is does not reach 1).

For ΔKSD-AR-LR sets A and B are used for producing Z values of area ratios for brother samples and *Z* values for non-brother samples. They are both regarded, respectively, for the numerator and denominator of the LR models according to the illustration in Fig. 3b. Then there are two more datasets required for generating *Z* values of area ratios for brother samples and *Z* values for non-brother samples for computing LR and testing its performance.

(c) set C, which is constructed likewise as in set A. These ΔKSD values are used for modeling the distributions when E and D are brothers and when they are not (black distributions in Fig. 3a, each composed of *b∑k*_*D*_ ΔKSD values);
(d) set D consisting of 2*Z* pairs of D and E samples (*Z* when E and D are brothers and *Z* when they are not), each pair with *k*_*D*_ X_f_ samples (Table 1), for computing 2*Z∑k*_*D*_ ΔKSD values. 2*Z* sets of *k*_*D*_ ΔKSD values each for 2*Z* pairs of E and D samples will be used for computing 2*Z* common areas ratios (AR) with distributions of set C when E and D are brothers and when they are not. The AR value for one of 2*Z* pairs of E and D samples is shown as a green line in Fig. 3b. The ARs are interpreted under H_1_ and H_2_ [distributions generated in (b)] to give LR. These LR values are used for estimating the levels of false positive answers (when LR should be lower than unity but it demonstrates values above 1), false negative rates (when LR should exceed unity but it is does not reach 1) and producing empirical cross entropy curves.

For ΔKSD-AR-LR there must be 2*b*+2*Z* pairs of brother D and E samples and 2*b*+2*Z* non-brother D and E samples. There is a limit of 210 pairs of brother D and E samples, thus *b* was arbitrarily set as 30, 50, 65, and *Z* as 40, so that it exploits the database quite efficiently (2∙65 + 2∙40 = 210). Test and training sets were developed *s* = 10 times for averaging results. For ΔKSD-AR model there must be *b* + *Z* pairs of brother D and E samples and *b* + *Z* non-brother D and E samples. The limit of 210 brother samples still holds, thus *b* was arbitrarily set as 60, 120, 170, and *Z* as 40 (170 + 40 = 210).

#### False positive and false negative answers

The performance of the proposed models was initially evaluated by estimating the levels of false positive responses for a set of *Z* non-brother samples and false negative responses for a set of *Z* brother samples, randomly selected in B set for ΔKSD-AR model and D set for ΔKSD-AR-LR model. False positive answers are observed when AR > 1 or LR > 1 for samples coming from different sources, which should yield AR < 1 or LR < 1. False negative answers are received when AR < 1 or LR < 1 for samples sharing the same origins, which should yield AR > 1 or LR > 1.

#### Empirical cross entropy approach

Nonetheless, validation of LR models solely through the prism of false response rates is an incomplete measure of performance, as it evaluates only the qualitative aspect of model functioning. It should be highlighted that the ability to discriminate between samples, however important, is not the only required characteristic of LR values set. Besides supporting the correct hypothesis, it is desired that the strength of this support is as high as possible for the particular proposition (i.e., LR >> 1 when H_1_ is correct and LR << 1 when H_2_ is correct). It is also crucial that the LR value provides weak support (LR value close to one) in case of rejecting the correct proposition. Only if both requirements are met it can be stated that the model effectively performs its function in the light of Bayesian theorem (Equation 4). Even if the model happens to support the incorrect hypothesis, it would deceive the representatives of justice only to a minor extent.

4 $$ \frac{\Pr \left({H}_1\right)}{\Pr \left({H}_2\right)}\cdot \frac{\Pr \left(E|{H}_1\right)}{\Pr \left(E|{H}_2\right)}=\frac{\Pr \left({H}_1|E\right)}{\Pr \left({H}_2|E\right)} $$

Empirical cross entropy (ECE) [11, 48, 49] is a procedure that allows the assessment of the qualitative and the quantitative aspect (strength of the support) of the model performance.

ECE is based on the idea of rewarding and penalizing the obtained LR values. The penalty is defined by *logarithmic strictly proper scoring rules* (if H_1_ is true: −log_2_(Pr(*H*_1_| *E*)), if H_2_ is true: −log_2_(Pr(*H*_2_| *E*))) and grows with stronger support for the incorrect hypothesis.

The ECE is a mean penalty weighted by the relevant prior probabilities Pr(H_1_) and Pr(H_2_):

5 $$ ECE=\frac{\Pr \left({H}_1\right)}{M_1}\sum \limits_{i\in 1}{\log}_2\left(1+\frac{\Pr \left({H}_2\right)}{LR_i\Pr \left({H}_1\right)}\right)+\frac{\Pr \left({H}_2\right)}{M_2}\sum \limits_{j\in 2}{\log}_2\left(1+\frac{LR_j\Pr \left({H}_1\right)}{\Pr \left({H}_2\right)}\right) $$

**Fig. 4:**
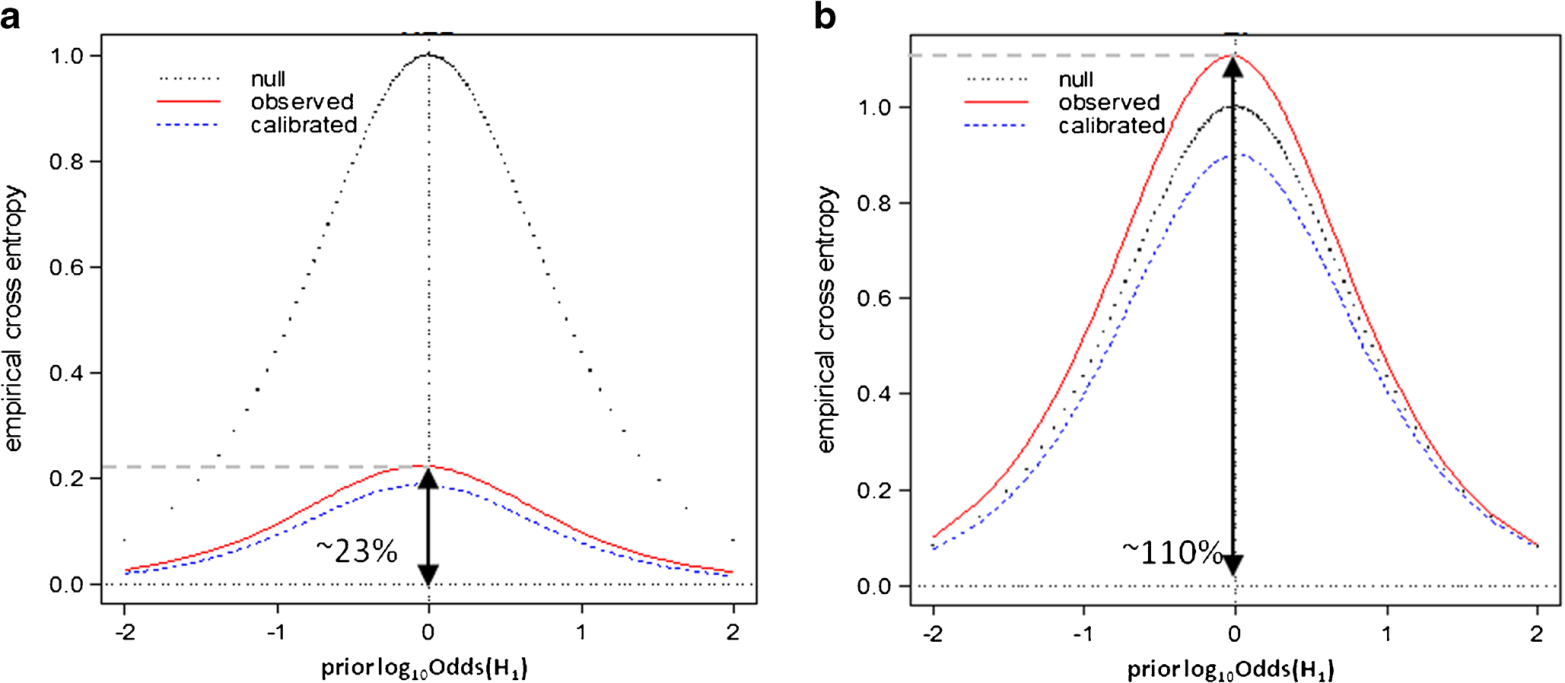
Empirical cross entropy (ECE) plots for LR models with **(a)** satisfactory, and **(b)** poor performance (detailed description in the text)

In general, the knowledge about a priori probabilities is not available because it can be acquired from a number of sources. For example, any specific knowledge about the person or company claiming the samples’ authenticity may serve as prior information. If the fact finder lacks the knowledge of the prior probabilities, or for the sake of objectivity, ECE for a set of all possible a priori probability quotients (prior odds) can be calculated and plotted. The ECE plot (Fig. 4) is composed of three components [49]:

- *Observed* curve (solid red) – represents the ECE values calculated in accordance with equation (5) for LR values subjected to the evaluation.
- *Calibrated* curve (dashed blue) – corresponds to the ECE values calculated for the LR values which have been transformed with the use of a pool adjacent violators (PAV) algorithm [48, 49]. The calibrated curve serves as an indicator of the LR values with the best performance of all LR sets that offer the same discriminating power.
- *Null* or *reference* curve (dotted black) – refers to the situation in which no evidential value is assigned to the data, i.e., LR = 1. Always being the same, the null curve should be treated as a reference.
  The performance of the chosen LR method can be evaluated through ECE plot analysis, where the *observed* curve can be assessed in terms of its position with respect to the *calibrated* and *null* curves. Figure 4 presents two ECE plots for LR models with satisfactory (Fig. 4a) and poor (Fig. 4b) performances. The arrows indicate how much information is unexplained by each model. In other words, they demonstrate the uncertainty about the correct hypothesis that remains when using particular LR model. For the satisfactory LR model the observed curve lies in between *calibrated* and *null* lines and points out some reduction of information loss. Such a reduction of information loss resulting from the employed LR method can be represented by the ECE value from the *observed* curve for the point of log_10_Odds(H_1_) = 0, which is referred to as ${C}_{llr}^{exp}$ value. Likewise, the value denoted as ${C}_{llr}^{\mathrm{min}}$ refers to the same point, but with respect to the *calibrated* curve. For the example shown in Fig. 4a, there is ca. 23% of information that is still unexplained by the model; hence, the reduction of information loss reaches 100% – 23% = 77%. For the LR model with poor performance, the *observed* curve exceeds the *null* curve, indicating that using such a model for data evaluation may end up in delivering more misleading information than when assuming that the data do not support any of the hypotheses (LR = 1 as in the null method illustrated by dotted black curve).

ECE approach was applied for controlling the performance of the LR-based model, i.e., ΔKSD-AR-LR. The ΔKSD-AR model is accomplished with the area ratio only, which just indicates which hypothesis is supported, but does not give the strength of the support towards the hypotheses.

### Software

The scripts were prepared in R programming language [50] using *pcaPP* and *MASS* packages.

## Results and discussion

### Descriptive statistics

The data matrix consists of concentrations of 46 elements (Mg, Ca, Sc, Ti, V, Cr, Mn, Fe, Co, Ni, Cu, Zn, Ga, As, Sr, Y, Zr, Nb, Mo, Ag, Cd, In, Sn, Sb, Ba, La, Ce, Pr, Nd, Sm, Eu, Gd, Tb, Dy, Ho, Er, Tm, Yb, Lu, Hf, Ta, Tl, Pb, Bi, Th, and U) in 5327 wolframite grains analyzed by LA-ICP-MS. In LA-ICP-MS, limits of detection (LOD) are obtained individually for each element in each grain and depend on the day to day performance of the instrument. For each element the results below LOD have been replaced by the median value of all element-specific LODs.

**Fig. 5:**
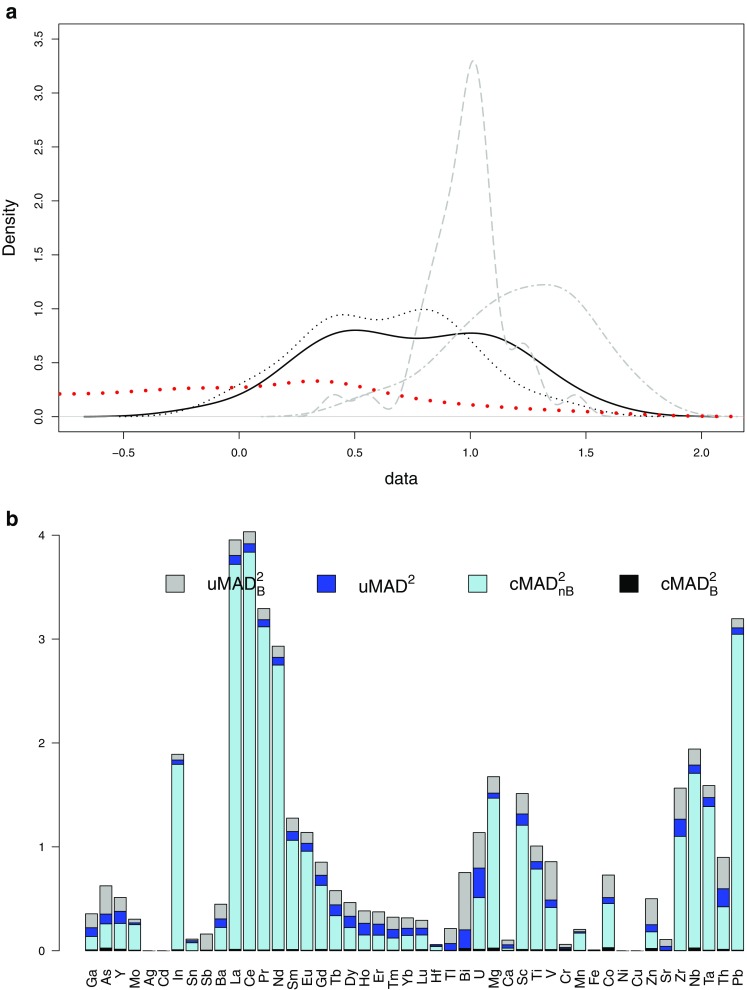
**(a)** The distribution of indium content (log-data) for the entire database (bolded red line) and for two pairs of brother samples (black and gray lines). **(b)** MAD2 computed for each element data (description can be found in the text). **(c)** MADB2 (the bottom bar) is so low that it is practically invisible in the plot

The element concentration data of single samples are not normally distributed according to the Shapiro-Wilk test (Fig. S2 in the ESM). Logarithmic transformation (ESM Fig. S2b) brings them a little bit closer to normality, but it still does not improve the situation significantly. A summary statistics of element concentrations in wolframite grains are given in Gäbler et al. 2017 [8]. Table 2 gives examples of distributions of element concentrations from different mine sites to illustrate the geochemical basis for sample discrimination. Figure 5a shows an example of the indium (log-data) distribution for four samples (two pairs of brother samples) and for the entire database. The plot clearly demonstrates that in spite of the similarity between brother samples, the significant data dispersion makes it difficult to differentiate between non-brother samples. This is typically observed when only single element content is studied. The differences between samples emerge only when more variables are considered at once.

**Table 2:** Examples of distributions of selected element concentrations in wolframite ore concentrates from different mine sites. Capital letters represent different mine sites. A1 and A2 represent two ore concentrates independently taken from the same mine site

Element	Zn [mg kg^-1^]			As [mg kg^-1^]			Lu [mg kg^-1^]			Pb [mg kg^-1^]		
Percentile	10^th^	50^th^	90^th^	10^th^	50^th^	90^th^	10^th^	50^th^	90^th^	10^th^	50^th^	90^th^
Rwanda												
A1	11	22	311	8.7	28.0	57.6	2.0	4.9	8.2	50.4	72.7	122.0
A2	7	13	151	14.1	35.4	94.1	0.9	5.2	9.4	44.5	70.5	110.0
B	16	25	109	153.1	546.6	2895.8	1.8	4.4	10.3	51.1	78.3	119.3
C	12	29	216	20.6	95.5	840.4	2.7	3.8	5.6	54.1	116.1	214.9
D	45	51	63	<0.3	<0.3	0.5	0.0	0.1	0.2	<0.2	0.9	2.9
DR Congo^a^												
E	67	113	156	<0.3	<0.3	<0.3	0.2	9.4	19.2	<0.2	<0.2	0.9
F	138	159	213	<0.3	<0.3	1.1	0.2	0.3	0.7	0.5	2.8	13.9
G	96	167	219	<0.3	<0.3	<0.8	3.5	8.3	18.8	<0.2	<0.2	<0.2
H	142	226	1375	0.3	0.5	1.9	0.3	0.4	0.9	3.0	6.8	20.0
Australia												
I	47	73	135	<0.3	12.2	162.6	68.9	186.2	423.6	0.4	20.6	291.4
K	124	137	159	<0.3	<0.3	<0.3	0.2	0.3	0.9	<0.2	0.5	1.3

^a^ Democratic Republic of the Congo

Various sources of dispersion of the log-data were studied using the robust measures, i.e., median and MAD^2^ (instead of mean and variance):

- *uMAD*^*2*^ - the within-samples MAD^2^ computed as a median of the MAD^2^ within each of the samples,
- *uMAD*_*B*_^*2*^ - the MAD^2^ within brother samples computed as a median of the MAD^2^ estimated within the sets combined of brother samples,
- *cMAD*_*nB*_^*2*^ - the MAD^2^ between non-brother samples (different mine sites) computed as the MAD^2^ of the medians representing each mine site,
- *cMAD*_*B*_^*2*^ - the MAD^2^ between brother samples computed as the median of the MAD^2^ of the medians representing each sample within each of the brother sample sets.

There were a few major observations regarding the estimated dispersion sources shown in Fig. 5b:

(i) The dispersion of data between brother samples, *cMAD*_*B*_^*2*^, is much lower than the dispersion between non-brother samples, *cMAD*_*nB*_^*2*^. This result is advantageous from the perspective of LR models, which easily differentiate non-brother samples and detect brother samples, when the similarity of the data observed for brother samples is greater than the similarity of the data for non-brother samples.
(ii) The within-samples dispersion, *uMAD*^*2*^, and dispersion within brother samples, *uMAD*_*B*_^*2*^, are comparable, but much greater than the variability between brother samples, *cMAD*_*B*_^*2*^ (which is hardly visible in the plot in Fig. 5b). This proves that the collective variability in the data for all of the brother samples is well reflected in the data recorded for a single sample. This is a promising statement, which confirms that despite brother samples being collected as separate samples from a single mine site, their data variability is still kept on the level observed for the grains collected as one sample. The latter observation is quite surprising and clearly points out huge variability of the data within each sample. Both *uMAD*^*2*^ and *uMAD*_*B*_^*2*^ are computed using all the measurements (i.e., grains) recorded for each sample. Contrary to that, *cMAD*_*B*_^*2*^ is estimated from the medians representing the measurements recorded for each sample. Thus the contribution of the data dispersion within each sample is not accounted for in *cMAD*_*B*_^*2*^. This is the reason for observing *cMAD*_*B*_^*2*^ lower than *uMAD*^*2*^ and *uMAD*_*B*_^*2*^.
(iii) The desired relation, i.e., lower dispersion of data between brother samples than between non-brother samples, is only observed when the samples are described by their medians, summarizing all measurements recorded for samples grains. Then the significant dispersion of these measurements is not accounted for and the non-brother samples become less similar than the brother samples.

Even though working with medians sounds like a solution to the problem, generalizing a sample’s data to a single number may be regarded as a loss of information. For this reason, the proposed LR models are constructed for pairs of samples instead of accounting for the entire database. Then the huge dispersion within each sample is easily managed using e.g., LDA. Another issue concerns lack of normality of the data within each sample and their multidimensionality, which should be sorted out to enable LDA. To handle these problems, rPCA was applied on the log-data to reduce data dimensionality by studying all variables at once. LDA was then used to find the direction that captures the differences between samples and is supposed to demonstrate greater similarity between brother samples than between non-brother samples. Finally the similarity between samples was expressed by Kolmogorov-Smirnov distances.

### Models performance

**Fig. 6:**
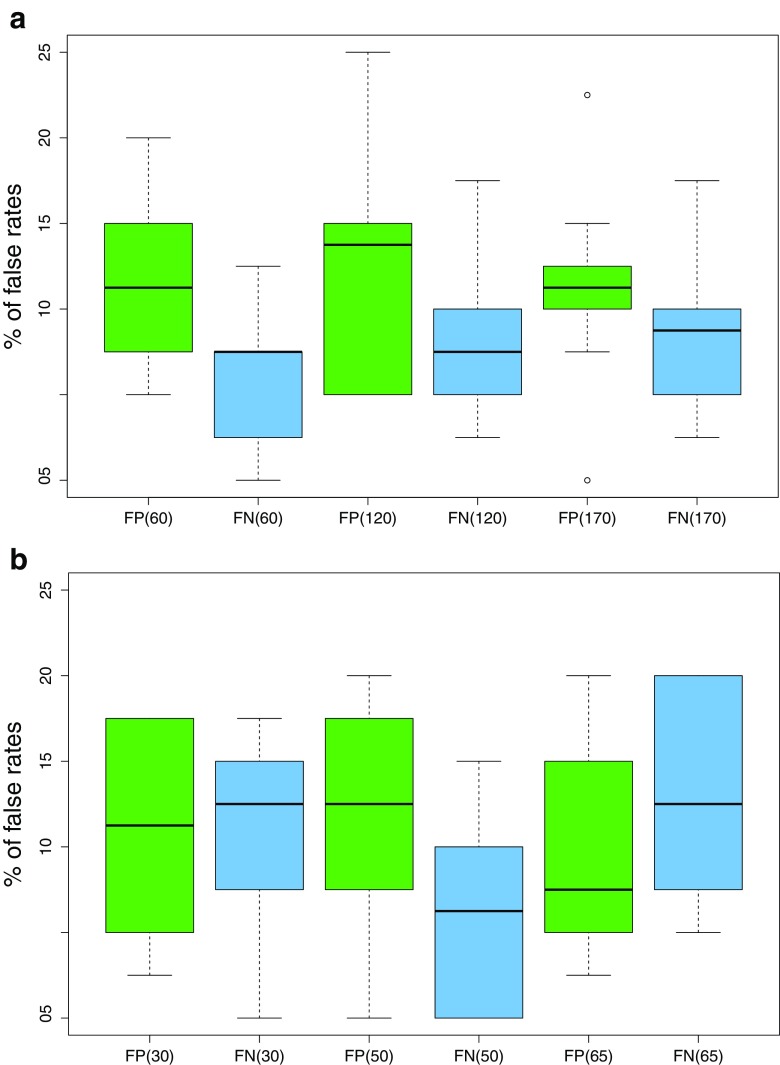
The levels of false positive (FP) and false negative (FN) model responses observed in *s* = 10 sets with respect to the number of pairs of E and D samples modeling the distributions for AR or LR calculations in **(a)** ΔKSD-AR, and **(b)** ΔKSD-AR-LR models

Figure 6 demonstrates the levels of false model responses with respect to the number of pairs of E and D samples modeling the distributions for AR or LR calculations, i.e., *b* pairs in sets A and C. Each boxplot is drawn from the outcomes generated in all *s* = 10 sets for averaging the results. It must be stressed that it becomes quite difficult to clearly indicate best behaving model. All models seem to yield acceptable outcomes with the levels of false positive and false negative responses usually oscillating up to 15%. The levels of misleading outcomes seem not to be affected by varying number of pairs of E and D samples modeling the distributions for AR or LR calculations (see labels under the boxplots in Fig. 6). This observation leads to the conclusions that the models are stable and deliver invariant results with respect to the number of samples used for modeling the distributions for AR or LR calculations. It enables receiving acceptable and reliable outcomes even using the small set for modeling the distributions under H_1_ and H_2_, which substantially saves computational time.

**Fig. 7:**
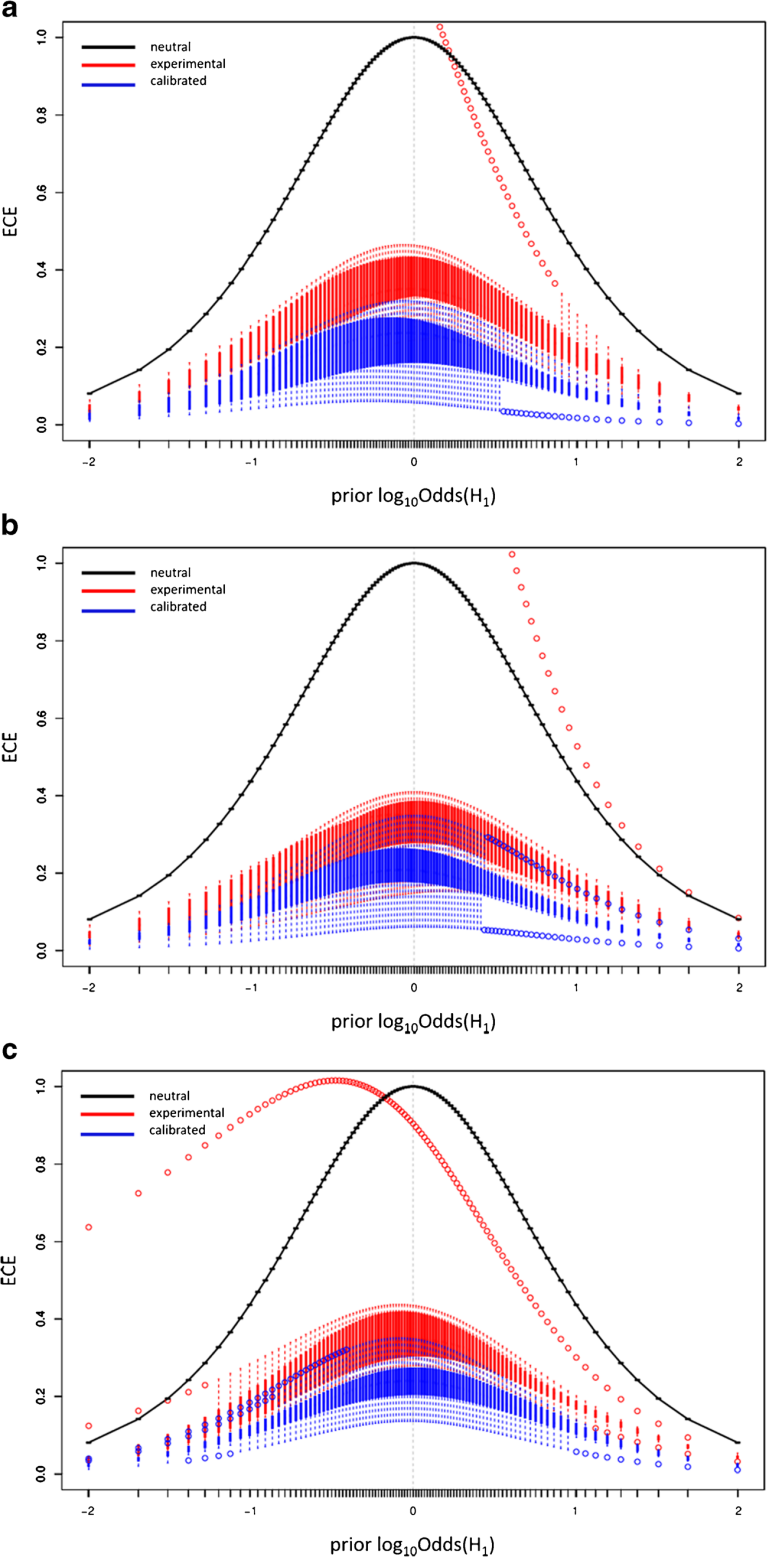
The ECE plots observed in *s* = 10 iterations for the ΔKSD-AR-LR model in regard to the number of pairs of D and E samples [**(a)** 30 pairs, **(b)** 50 pairs, **(c)** 65 pairs] modeling the distributions for LR calculations

Figure 7 illustrates the empirical cross entropy (ECE) plots for the ΔKSD-AR-LR model accomplished with LR computations in regard to the number of pairs of E and D samples modeling the distributions for LR calculations. The diagrams portray the empirical cross entropy plots in a modified way in comparison to traditional ECE curves as introduced above. The experimental and calibrated curves are replaced by the sets of boxplots accounting for all ECE values calculated in *s* = 10 sets. Thus, for each quotient of the prior odds the boxplot is drawn from all *s* = 10 sets. ECE plots clearly indicate that ΔKSD-AR-LR models explain a large part of the information in the data; however, they sometimes introduce misleading information. There is no remarkable improvement of the ECE plots appearance with growing number of pairs of E and D samples taken for modeling the distributions for LR calculations.

The undesirable shape of the ECE curves, which go beyond the neutral (null) curve for some ranges of the logarithm of the prior odds, i.e., log_10_Odds(H_1_), was studied in-depth in order to determine whether this model truly yields poor performance, or this statement is just exaggerated as it may be caused by only a single sample delivering strong misleading support towards the incorrect hypothesis. It appears that in most cases the deteriorated curvature of the ECE plots is the consequence of generating only few LR values that support the incorrect hypothesis (usually H_2_) much stronger than the remaining values support the correct hypothesis (usually H_1_). This drawback of the ECE plots forces the researcher to be careful when the performance of the models assessed by ECE approach appears to be poor.

Observable differences between the experimental (known also as observed) and calibrated curves point out that there still exist some opportunities for developing the proposed methodology for receiving more reliable outcomes.

**Fig. 8:**
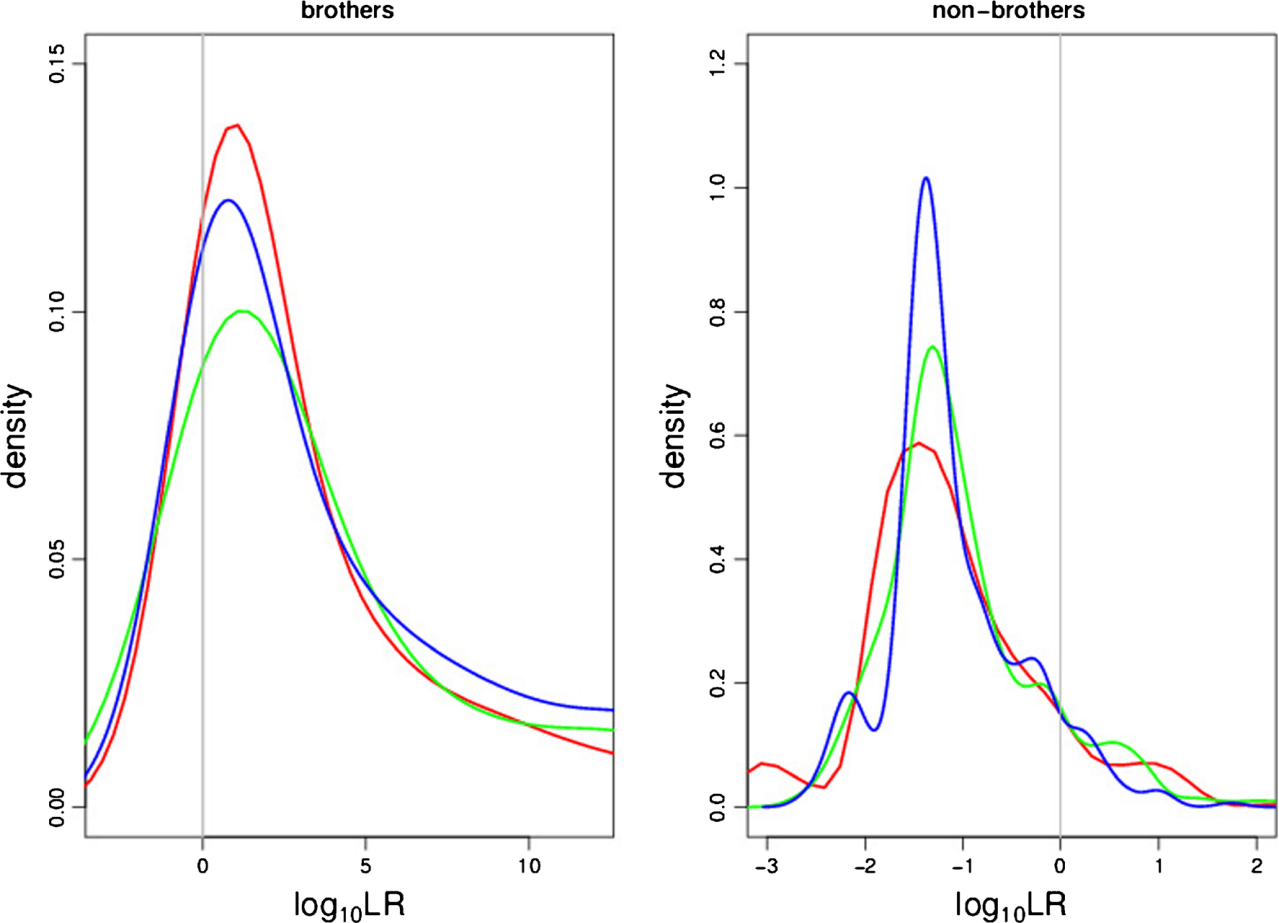
The distribution of log_10_LR values observed in model ΔKSD-AR-LR in regard to varying number of samples (30, 60, 75 marked in different colors) modeling the distributions under H_1_ and H_2_. Each distribution refers to all *s* = 10 sets, i.e., 400 LR values

Figure 8 shows the distribution of log_10_LR values received for brothers (left) and non-brothers (right) for all developed three variants of the model ΔKSD-AR-LR (serving as an example) involving 30, 50, 65 pairs of E and D samples generating the distributions. Each distribution refers to the 40 LR values between brothers or non-brothers received in all *s* = 10 sets, which is in total 400 LR values. The plots confirm previous observations that the models are insensitive to the varying number of pairs of E and D samples modeling the distributions. This favorable remark leads to the conclusion that the developed models are stable and are not subject to parameters fluctuations easily.

### Casework example

The performance of the proposed ΔKSD-AR and ΔKSD-AR-LR models is shown for two casework examples:

(i) Five samples from the wolframite trading chain with reliable source documents (origin M) were used as evidence samples E. The database comprises nine reference samples from mine site M which were regarded as D samples. The arising question was whether E samples really came from the declared source (mine site M). Put in other words, whether E and D were brother samples (H_1_) or not (H_2_). To answer this query, there were 9∙5 = 45 pairs of E and D samples tested. Since they are labeled as brother samples (H_1_), they are supposed to deliver LR or AR greater than 1.
(ii) For comparison, with the results obtained from (i) 45 non-brother pairs of samples coming from two different mine sites were selected by chance from the database. One sample of each pair was treated as sample E, the other one as sample D. LR or AR below 1 (H_2_) were expected for these comparisons.

Both developed models are applied on the casework data. Unfortunately, they cannot be directly compared with regard to the strength of the support towards the hypotheses. This is a consequence of the fact that LR value is not obtained by the ΔKSD-AR model, contrary to the ΔKSD-AR-LR model.

**Fig. 9:**
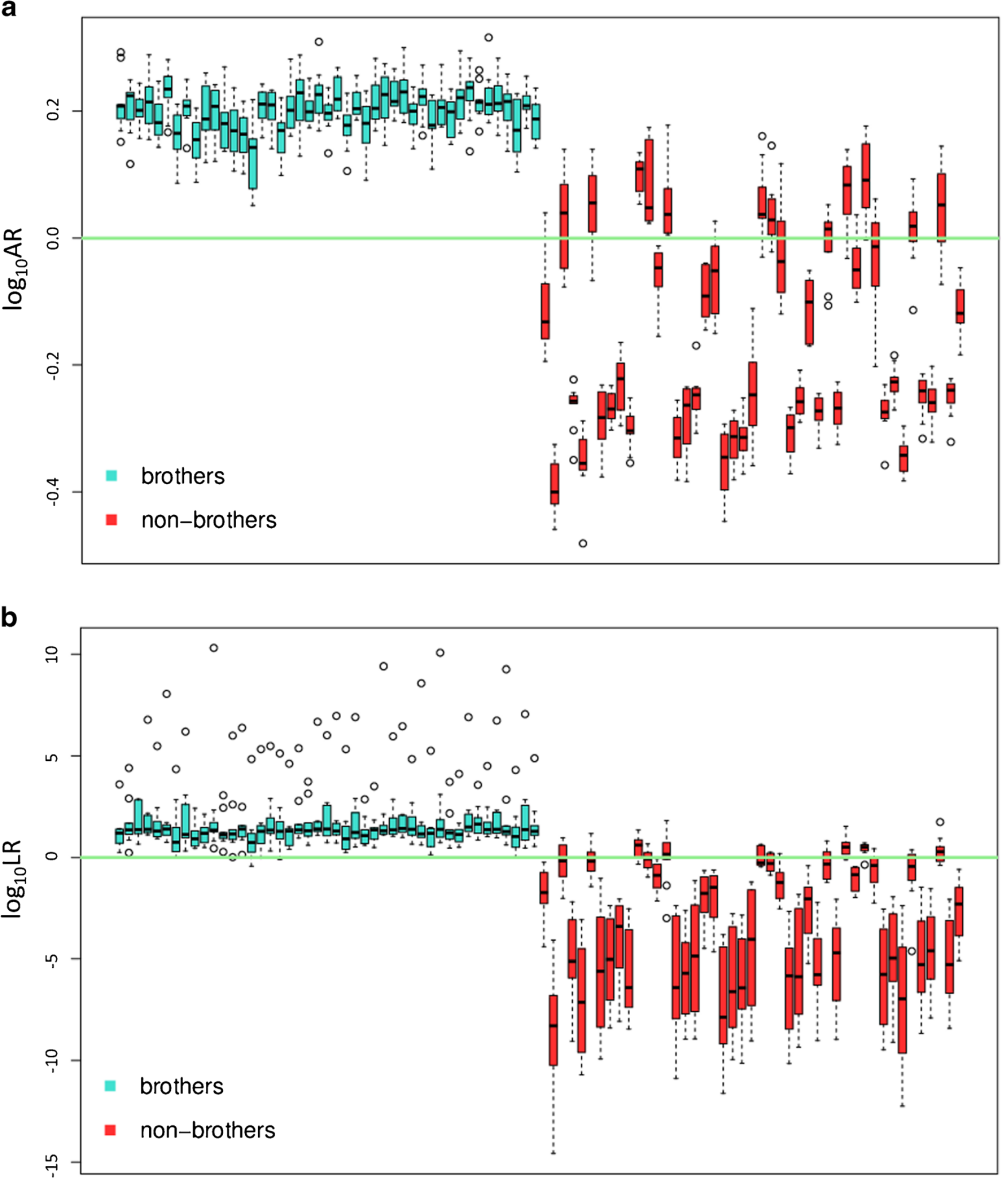
**(a)** log_10_AR, and **(b)** log_10_LR values observed for an example casework in model ΔKSD-AR and ΔKSD-AR-LR, respectively. Each boxplot (blue for brothers and red for non-brothers) refers to 10 outcomes computed for averaging the results. Green horizontal line represents the threshold for decision making (log_10_AR or log_10_LR = 0)

The results are illustrated in the form of boxplots given in Fig. 9 showing the sets of log_10_LR or log_10_AR values for each pair of E and D samples generated in 10 iterations for averaging the outcomes. The AR or LR values for individual calculations of the same pair of samples E and D cannot be expected to be identical. This is because the brother and non-brother pairs which are selected from the database to construct the distributions typical for brothers and non-brother sample pairs vary for each calculation.

The area ratios (AR) obtained for the sample pairs in case (i) are all above 1 (or 0 on the log scale) and oscillate around 2. The ΔKSD-AR-LR model supports the H_1_ quite strongly, though there are a few false negative responses. They, however, support the incorrect hypothesis only moderately and are therefore rather incidental.

For non-brother pairs in case (ii) hypothesis H_2_ is supported for the majority of the non-brother pairs, but there are a few outcomes observed that misleadingly suggest that the samples originate from the same mine site, although they truly come from different sources. However, it is observable as well that these results for the ΔKSD-AR-LR model do not support the incorrect hypothesis H_1_ strongly and that the support is comparable to the support for the incorrect hypothesis H_2_ generated for brother samples.

This example clearly illustrates that the models place an emphasis on minimizing the levels of false negative answers considered when the samples are brothers. This seems quite important for real casework, where accusing a person or company of declaring the wrong origin of a wolframite delivery in a situation when the declared origin is actually true, should always be avoided. Conversely, the reverse situation, when the fact finder is deceived about the origins of wolframites, has no legal consequences and simply allows the deception to go undetected in that instance. For this reason, the levels of false negative rates must be strictly controlled while it is acceptable for the levels of false positive answers to be slightly greater.

## Conclusions

The research presented herein addresses the issue of verifying the authenticity of the declared origins of wolframite samples based on their elemental composition determined by LA-ICP-MS. In the case of a database with multivariate data, huge dispersion of the samples, and clearly not-normal distribution of the data, the evaluation of the evidential value can be supported by using hybrid likelihood ratio models that take the best from the chemometric tools and smartly apply the results within the LR framework. The robust PCA and LDA used in this study are applied to efficiently reduce data dimensionality and extract the features that maximally differentiate between samples coming from different mine sites (non-brother samples). A score-based LR model that incorporated similarity metrics like the Kolmogorov-Smirnov distance (KSD) into the likelihood ratio approach was developed to conclude whether a sample in question with a declared origin and a reference sample (truly coming from the declared location) are brother samples or not.

Two models called ΔKSD-AR and ΔKSD-AR-LR were proposed. The ΔKSD-AR model used the ratio of the common areas of distributions of similarity metrics found for the sample in question (E) compared with its reference sample (D) and typical brother or non-brother samples, respectively. The ΔKSD-AR-LR model extended this model by coupling it with the likelihood ratio approach. Then it was possible not only to conclude which hypothesis was supported (as in ΔKSD-AR model), but also to express the strength of such support.

Both models deliver acceptable results with false positive and false negative rates oscillating around 10%–15%. ΔKSD-AR-LR model significantly reduces information loss expressed by the empirical cross entropy curves. The only drawback of the ΔKSD-AR model relates to its accomplishment with the ratio, which cannot be treated directly as LR. The advantage of the ΔKSD-AR-LR model is the fact that its performance can be objectively assessed by the ECE approach stressing the magnitude of the support towards each of the hypotheses. In a casework example, both models were tested successfully, confirming the brother nature of reliable samples from the trading chain relative to their respective reference samples.

The evaluation of the models performance indicates that the levels of false negative rates are minimized in regard to the false positive rates. This allows for avoiding the situation in which true declared origins of samples are regarded as spurious and the declaring person or company is recognized as a liar. This remains in contrast to the typical forensic issues where an emphasis is put on lowering the levels of false positive rates, leading to accusation of an innocent person or company. This is because in the wolframites case innocence means finding two samples supporting the H_1_ (stating that they come from the same source), whilst in the forensic science innocence involves finding e.g., two pieces of evidence as coming from different sources, hence in support for the H_2_.

The proposed models have been developed for the conflict mineral wolframite. They also work for other minerals that are traded as ore concentrates like coltan or cassiterite because, just like wolframite, those minerals are not chemically modified at the mine site and keep their chemical signature during trade down to the smelter/metal refinery. The application of the proposed models on minerals like heterogenite or gold, which are often chemically modified at the mine site, seems to be more difficult as the chemical modification might change the characteristic geochemical signature of the mined ore.

## Electronic supplementary material

ESM 1 (PDF 466 kb)
