# Supplementary material for: Geochemical wolframite fingerprinting – the likelihood ratio approach for laser ablation ICP-MS data
Source: Anal Bioanal Chem. 2018 Apr 17;410(13):3073–91. doi: 10.1007/s00216-018-1007-9 (PMC5910492; doi:10.1007/s00216-018-1007-9)
Supplement: Supplementary file 1 — (PDF 466 kb) [file 216_2018_1007_MOESM1_ESM.pdf]

Analytical and Bioanalytical Chemistry

Electronic Supplementary Material

Geochemical wolframite fingerprinting – the likelihood ratio approach for laser ablation ICP-MS data

Agnieszka Martyna, Hans-Eike Gäbler, Andreas Bahr, Grzegorz Zadora

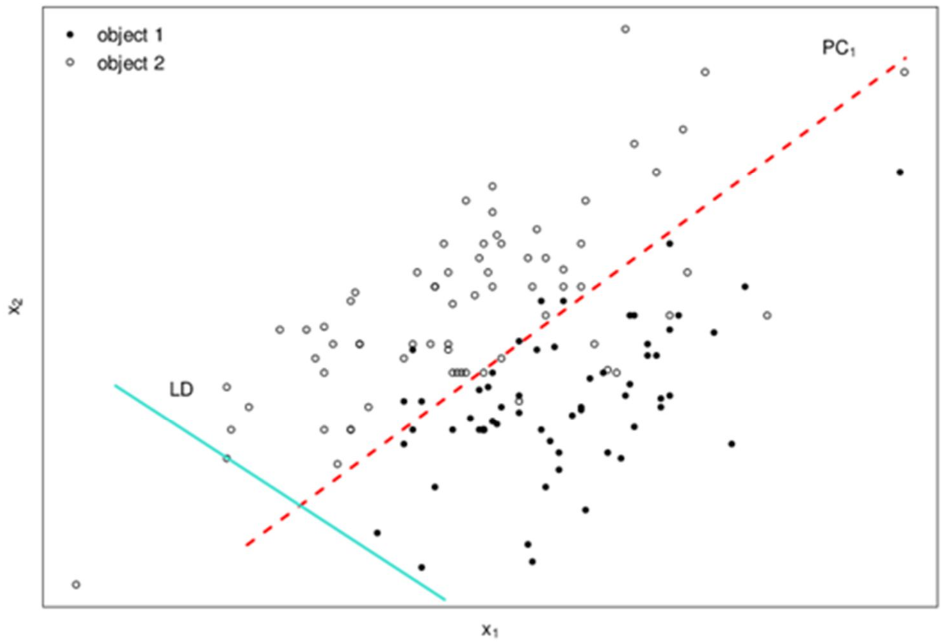

Fig. S1 The illustration of the differences between variability components addressed by PCA and LDA for two groups of data marked by full and empty circles

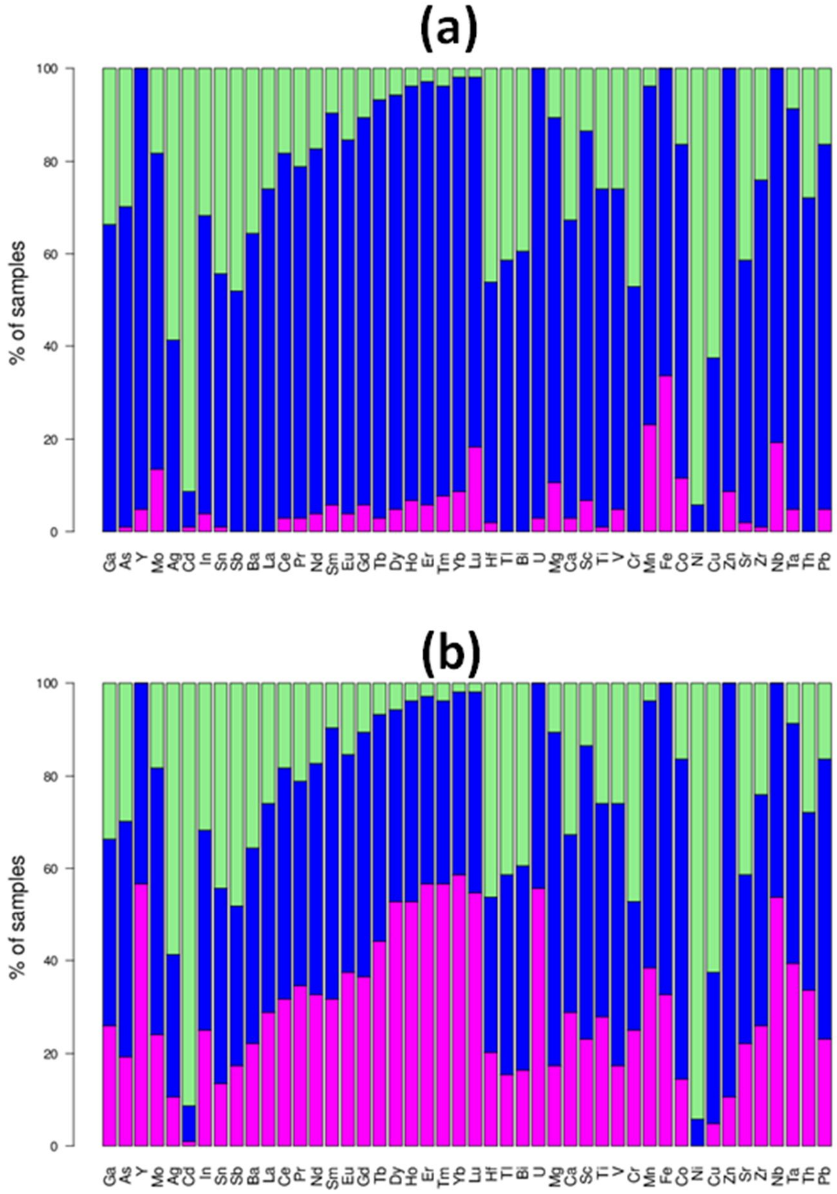

Fig. S2 The results of Shapiro-Wilk test for normality showing in purple the percentage of samples demonstrating normal distribution (a) before and (b) after the logarithmic transformation. Blue bars refer to the proportion of samples with not normal distribution and green bars relate to the samples where for more than half of grains the measurements were below detection limits
